# Aging Is a Risk Factor for Utricular Dysfunction in Idiopathic Benign Paroxysmal Positional Vertigo

**DOI:** 10.3389/fneur.2018.01049

**Published:** 2018-12-03

**Authors:** Chisato Fujimoto, Takuya Kawahara, Makoto Kinoshita, Yayoi S. Kikkawa, Keiko Sugasawa, Masato Yagi, Tatsuya Yamasoba, Shinichi Iwasaki, Toshihisa Murofushi

**Affiliations:** ^1^Department of Otolaryngology, Tokyo Teishin Hospital, Tokyo, Japan; ^2^Department of Otolaryngology and Head and Neck Surgery, Graduate School of Medicine, University of Tokyo, Tokyo, Japan; ^3^Biostatistics Division, Clinical Research Support Center, The University of Tokyo Hospital, Tokyo, Japan; ^4^Department of Otolaryngology, School of Medicine, Teikyo University Mizonokuchi Hospital, Kawasaki, Japan

**Keywords:** aging, benign paroxysmal positional vertigo, risk factors, vestibular diseases, vestibular function tests

## Abstract

Benign paroxysmal positional vertigo (BPPV) is the most common cause of balance disorders in the elderly. Dislodgement of the otoconia in BPPV might have an association with damage to the otolith organs. The aim of this study was to investigate whether aging is a risk factor for otolith organ dysfunction in idiopathic BPPV. We retrospectively reviewed the medical records of 112 consecutive idiopathic BPPV patients who underwent cervical VEMP testing to air-conducted sound (ACS cVEMP), ocular VEMP testing to bone-conducted vibration (BCV oVEMP), and caloric testing. We performed binomial logistic regression analyses to see whether age, the side affected by BPPV or the canal affected by BPPV have an association with the presence of peripheral vestibular dysfunction in idiopathic BPPV patients. The elderly group (aged ≥65 years) had a significantly positive association with abnormalities in BCV oVEMPs (*p* = 0.0109), while the side affected by BPPV (*p* = 0.598) and the canal affected by BPPV (*p* = 0.576) did not. The odds ratio of the abnormal BCV oVEMPs for the elderly group compared with the non-elderly group (aged < 65 years) was 2.676 (95% confidence interval, 1.254–5.079). The elderly group had no significant association with the abnormalities in ACS cVEMPs (*p* = 0.0955) or caloric testing (*p* = 0.488). Dysfunction of the utricle, where the dislodgement of the otoconia mainly occurs, is affected by aging in idiopathic BPPV.

## Introduction

Benign paroxysmal positional vertigo (BPPV) is the most common disease that causes vertigo (1). It is also the most common cause of balance disorders in the elderly (2, 3). The 1-year prevalence of BPPV increases with age and it is ~7 times higher in the age group above 60 years old than in the 18–39 year age groups (4). Generally, some BPPV patients will resolve spontaneously without treatment, and others can be cured by physical therapy. However, elderly BPPV patients show a more prolonged clinical course than younger adult patients (5). Elderly BPPV patients often report chronic dizziness instead of positional vertigo (6), and BPPV with chronic dizziness is often under-recognized. This might be one of the reasons for the prolonged clinical course. Additionally, elderly BPPV patients show less improvement from physical therapy and a higher recurrence frequency (5).

BPPV is considered to arise from dislodgement of particulate matter, probably fragments of otoconia from the otolith organ, mainly from the utricle, and the migration of this matter into the semicircular canals (SCCs) (7). Dislodgement of the otoconia in BPPV might have an association with damage to the otolith organs. A histopathological study of temporal bones in BPPV showed that the otolith macula was severely damaged (8). Additionally, BPPV is known to arise secondarily to inner ear disorders such as vestibular neuritis, Meniere's disease, and sudden sensorineural hearing loss, as well as traumatic events such as head trauma (9). The mechanisms of otoconia dislodgement in these cases of BPPV are assumed to result from damage to the otolith macula by mechanical stimuli, viral lesions, or a vascular compromise of the vestibular artery. In addition, BPPV has been shown to be related to abnormalities in otolith function tests (10–12). Some reports showed that abnormal findings in vestibular evoked myogenic potential (VEMP) testing, the most widely used test of otolith function, were found on the side affected by BPPV (10, 12). On the other hand, another report showed that not only the affected side but also the unaffected side in the BPPV group showed significantly higher abnormalities in VEMPs compared with the healthy control group (11). This paper also revealed that VEMP abnormalities persisted even after successful physical therapy, suggesting that otolith organ dysfunction would remain after recovery from BPPV (11).

The number of hair cells in the otolith organs decreases with age in humans (13). Additionally, the abnormalities in VEMPs increases with age in healthy individuals (14–16). These findings suggest that the otolith organs are morphologically and functionally damaged by aging. In the present study, we investigated whether aging is a risk factor in otolith organ dysfunction in idiopathic BPPV patients. We revealed that the elderly group (aged ≥65 years) had a significant positive association with utricular dysfunction detected by ocular VEMP (oVEMP) testing, but it had no significant association with saccular dysfunction detected by cervical VEMP (cVEMP) testing or lateral SCC (LSCC) dysfunction detected by caloric testing. Our findings imply that BPPV is common in the elderly due to the increased likelihood of utricular damage which leads to a more frequent dislodgement of the otoconia.

## Materials and Methods

### Study Design

This study was approved by Research Ethics Committee, Graduate School of Medicine and Faculty of Medicine, at the University of Tokyo (2487) and was conducted according to the tenets of the Declaration of Helsinki. Written informed consent was waived because of the retrospective design.

### Subjects

We retrospectively reviewed the medical records of 112 consecutive idiopathic BPPV patients who underwent cVEMP testing to air-conducted sound (ACS cVEMP), oVEMP testing to bone-conducted vibration (BCV oVEMP), and caloric testing between January 2009 and May 2018 at the Balance Disorder Clinic of the University of Tokyo Hospital. Caloric testing has been used to clinically assess the function of the lateral semicircular canal (LSCC) and superior vestibular nerve. ACS cVEMP testing has been used to clinically assess the function of the saccule and inferior vestibular nerve (17–19). BCV oVEMP testing has been used to evaluate the function of the utricle and the superior vestibular nerve (19–21). We used the diagnostic criteria of BPPV, which was formulated by the Committee for Classification of Vestibular Disorders of the Bárány Society (22). Positional nystagmus was monitored using an infrared charge-coupled device camera.

### ACS cVEMP Testing

The recording methods for ACS cVEMPs have been previously described (23). Surface electromyographic (EMG) electrodes were placed on the upper half of each sternocleidomastoid muscle (SCM), with a reference electrode placed on the lateral end of the upper sternum. During the recording procedure, the subjects were asked to lie in a supine position and raise their heads off the bed to contract the SCM. Neuropack R (Nihon Kohden Co. Ltd., Tokyo, Japan) was used to record the cVEMPs. The acoustic stimuli consisted of air-conducted 500 Hz short tone bursts (135 dBSPL, rise/fall time 1 ms, plateau time 2 ms) presented through headphones at a stimulation rate of 5 Hz. The signals were amplified and bandpass filtered (20–2,000 Hz). The analysis time was 100 ms. The background EMG of the subject was monitored during recording to confirm that the subject's SCM activity was adequately maintained at a sufficient level (150 μV). Two runs were performed for each ear to confirm the reproducibility of the test. The amplitude and latency of the first positive–negative peak (p13–n23) was obtained from the average of 2 responses. The p13-n23 amplitude recorded from the side ipsilateral to stimulation was analyzed. An asymmetry ratio for cVEMPs (cVEMP AR) was used to evaluate any abnormality in the p13-n23 amplitude (24). An “absent response” was noted when a reproducible p13–n23 was not observed. A “decreased response” was noted when the cVEMP AR was greater than the normal upper limit, which was set at 34.0 (24). A patient who showed absent cVEMP responses on both sides in spite of sufficient muscle contraction was regarded as having bilaterally abnormal cVEMPs. The mean (±SD) latency of p13 in ACS cVEMPs in normal subjects has been reported as 14.9 (±0.53) ms (24). An abnormal latency was noted if the p13 latency was outside the normal range (mean ± 2 SD). One BPPV patient who showed an air-bone gap (>10 dB) in pure tone audiometry was excluded from the analyses in the present study.

### BCV oVEMP Testing

The recording methods for BCV oVEMPs have been previously described (23). Surface electrodes were placed on the skin 1 cm below (active) and 3 cm below (reference) the center of each lower eyelid. During the recording procedure, the subjects were asked to lie in a supine position and to look up by ~30 degrees. Neuropack R was used to record the oVEMPs. The bone-conducted stimuli consisted of 500 Hz tone-bursts (rise/fall time 1 ms, plateau time = 2 ms) delivered by a 4,810 mini-shaker (Bruel and Kjaer, Naerum, Denmark), which was placed on the forehead in the midline (Fz), at a stimulation rate of 3 Hz. The driving voltage was adjusted to 8.0 V (peak to peak), and it produced a peak force level of 128 dB re 1 mN. The signal was amplified and bandpass-filtered (0.5–500 Hz). The analysis time was 50 ms. Two runs were performed to confirm the reproducibility of the test. The amplitude and latency of the first negative–positive peak (n1–p1) was obtained from the average of 2 responses. The n1–p1 amplitude recorded from the side contralateral to stimulation was used for the analyses. The oVEMP asymmetry ratio (oVEMP AR) was used to evaluate the abnormality of the n1–p1 amplitude (25). An “absent response” was noted when a reproducible n1–p1 was not observed. A “decreased response” was noted if the oVEMP AR was greater than the normal upper limit, which was set at 27.3 (26). A patient who showed absent oVEMP responses on both sides was regarded as having bilaterally abnormal oVEMPs. The mean (±SD) latency of n1 for BCV oVEMP in normal subjects has been reported as 10.4 (±0.63) ms (27). An abnormal latency was noted if the n1 latency was outside the normal range (mean ± 2 SD).

### Caloric Testing

Caloric testing was conducted by irrigating the external auditory canal with 2 ml ice water for 20 s; the induced nystagmus was recorded using electronystagmography in a darkened room. This ice-water caloric testing is easier to conduct than bithermal caloric testing and shows a high sensitivity and specificity for the detection of canal paresis (CP) based on Jongkee's formula (28). An abnormal response was noted when a patient fulfilled either of the following criteria: (1) CP percentage >20% (29); (2) maximum slow phase eye velocity of caloric nystagmus < 10 degrees/s bilaterally (30).

### Data Analysis

Statistical analyses were conducted by using SAS software version 9.4 (SAS Inc., Cary, NC, USA). Binomial logistic regression analyses were performed to see whether the subjects' age, the side affected by BPPV or the canal affected by BPPV have an association with the presence of peripheral vestibular dysfunction in idiopathic BPPV patients. The elderly group were defined as aged 65 years and over, and the non-elderly group were defined as younger than 65 years. The dependent variables were abnormalities in the results of caloric, ACS cVEMP, or BCV oVEMP testing, and the independent variables were age (the non-elderly group vs. the elderly group), the side affected by BPPV (the unaffected side vs. the affected side) and the canal affected by BPPV (the posterior SCC (PSCC) vs. the LSCC). *p* < 0.05 was considered statistically significant without adjustment for multiple testing.

## Results

**Figure 1:**
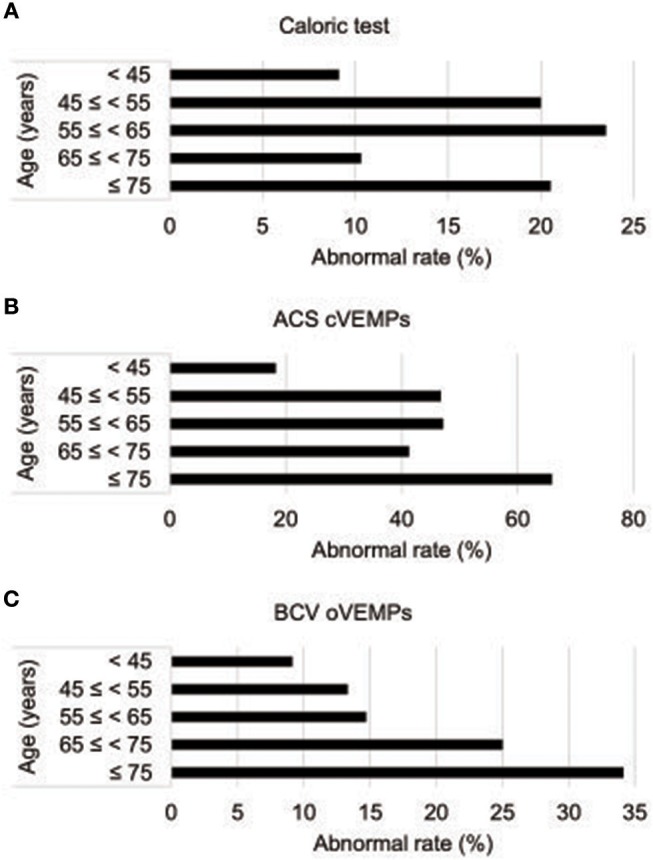
Abnormality rates in vestibular function tests by age **(A)** Abnormality rate in caloric tests by age. **(B)** Abnormality rates in ACS cVEMPs by age. **(C)** Abnormality rates of BCV oVEMPs by age. ACS cVEMPs, cervical vestibular evoked myogenic potentials to air-conducted sound; oVEMPs, ocular vestibular evoked myogenic potentials to bone-conducted vibration.

Of the 112 idiopathic BPPV patients who underwent caloric, ACS cVEMP and BCV oVEMP testing, one patient showed an air-bone gap in pure tone audiometry. We excluded this patient from the analysis. We excluded 10 LSCC BPPV patients from the analysis because their affected side was undetected. We also excluded one patient with anterior SCC BPPV (ASCC BPPV) and one patient who had both the LSCC and PSCC affected. Therefore, we analyzed the data obtained from the remaining 99 patients (32 males and 67 females; mean [±standard deviation] age, 63.0 [±14.2] years; range, 20–89 years) (Table 1). Out of the 99 idiopathic BPPV patients, 28 (28%) showed abnormal caloric responses, 60 (61%) had abnormal cVEMPs, and 30 (30%) had abnormal oVEMPs (Table 2). Out of 29 patients with unilateral abnormal ACS cVEMPs, 15 showed unilateral absent responses, 12 showed unilateral decreased responses, and 2 showed unilateral long latencies. Out of 17 patients with unilateral abnormal BCV oVEMPs, 8 showed unilateral absent responses and 9 showed unilateral decreased responses. Three patients showed absent ACS cVEMP and BCV oVEMP responses in one side. As the severity of vestibular dysfunction increased, as measured by cVEMPs and oVEMPs, the proportion of elderly patients tended to increase. The rate of abnormal oVEMPs increased with age (Figure 1).

**Table 1:** Characteristics of 99 idiopathic BPPV patients included for the analyses.

**Characteristics**	**No. (%)**
**AGE**	
Non-elderly group (<65years)	43 (43%)
Elderly group (65 years and over)	56 (57%)
**SEX**	
Male	32 (32%)
Female	67 (68%)
**SIDE OF AFFECTED EAR**	
Left	42 (42%)
Right	57 (58%)
**CANAL AFFECTED BY BPPV**	
PSCC	59 (60%)
LSCC	40 (40%)

*BPPV, benign paroxysmal positional vertigo; SD, standard deviation; PSCC, posterior SCC; LSCC, lateral SCC*.

**Table 2:** Characteristics of vestibular function in 99 idiopathic BPPV patients included for the analyses.

		**Characteristics**		
	**No**.	**Elderly group No. (%)**	**Affected side No. (%)**	**LSCC BPPV No. (%)**
**CALORIC TEST**				
Bilaterally abnormal	4	4 (100%)	–	2 (50%)
Unilaterally abnormal	24	8 (33%)	15 (63%)	12 (50%)
Normal	71	44 (62%)	–	26 (37%)
**ACS cVEMPs**				
Bilaterally abnormal	31	20 (65%)	–	16 (52%)
Unilaterally abnormal	29	17 (59%)	15 (52%)	7 (24%)
Normal	39	19 (49%)	–	17 (44%)
**BCV oVEMPs**				
Bilaterally abnormal	13	11 (85%)	–	4 (31%)
Unilaterally abnormal	17	10 (59%)	7 (41%)	7 (41%)
Normal	69	35 (51%)	–	29 (42%)

*BPPV, benign paroxysmal positional vertigo; LSCC, lateral semicircular canal; ACS cVEMPs, cervical vestibular evoked myogenic potentials to air-conducted sound; BCV oVEMPs, ocular vestibular evoked myogenic potentials to bone-conducted vibration*.

First, we investigated whether age, the side affected by BPPV or the canal affected by BPPV had an association with abnormalities in caloric testing in idiopathic BPPV (Table 3). Logistic regression analysis showed that there was no significant association [*p* = 0.488 (age), *p* = 0.247 (side affected by BPPV), *p* = 0.261 (canal affected by BPPV)]. The odds ratio of the abnormal caloric responses for the elderly group compared with the non-elderly group, for the affected side compared with the unaffected side, and for the LSCC BPPV compared with PSCC BPPV was 0.762 (95% confidence interval (CI), 0.354–1.642), 1.578 (95% CI, 0.729–3.416), 1.553 (95% CI, 0.721–3.348), respectively.

**Table 3:** Binomial logistic regression analyses for investigating the association between the abnormalities in caloric testing and age, the side affected by BPPV and the canal affected by BPPV in idiopathic BPPV patients.

**Variable**	**Odds ratio (95% CI)**	***p*-value**
**AGE**		
Elderly group	0.762 (0.354–1.642)	0.488
Non-elderly group	Reference
**SIDE AFFECTED BY BPPV**		
Affected side	1.578 (0.729–3.416)	0.247
Unaffected side	Reference
**CANAL AFFECTED BY BPPV**		
LSCC	1.553 (0.721–3.348)	0.261
PSCC	Reference

*BPPV, benign paroxysmal positional vertigo; SCC, semicircular canal; SE, standard error; CI, confidence interval; PSCC, posterior SCC; LSCC, lateral SCC*.

Next, we investigated whether age, the side affected by BPPV or the canal affected by BPPV had an association with abnormalities in ACS cVEMP testing in idiopathic BPPV (Table 4). Logistic regression analysis showed that there were no significant associations [*p* = 0.0955 (age), *p* = 0.886 (side affected by BPPV), *p* = 0.405 (canal affected by BPPV)], although the elderly group tended to show abnormal ACS cVEMPs. The odds ratio of abnormal ACS cVEMPs for the elderly group compared with the non-elderly group, for the affected side compared with the unaffected side, and for LSCC BPPV compared with PSCC BPPV was 1.631 (95% CI, 0.918–2.897), 1.042 (95% CI, 0.593–1.831), 1.278 (95% CI, 0.717–2.278), respectively.

**Table 4:** Binomial logistic regression analyses for investigating the association between abnormalities in ACS cVEMP testing and age, the side affected by BPPV and the canal affected by BPPV in idiopathic BPPV patients.

**Variable**	**Odds ratio (95% CI)**	***p*-value**
**AGE**		
Elderly group	1.631 (0.918–2.897)	0.0955
Non-elderly group	Reference
**SIDE AFFECTED BY BPPV**		
Affected side	1.042 (0.593–1.831)	0.886
Unaffected side	Reference
**CANAL AFFECTED BY BPPV**		
LSCC	1.278 (0.717–2.278)	0.405
PSCC	Reference

*ACS cVEMP, cervical vestibular evoked myogenic potential to air-conducted sound; BPPV, benign paroxysmal positional vertigo; SCC, semicircular canal; SE, standard error; CI, confidence interval; PSCC, posterior SCC; LSCC, lateral SCC*.

We then investigated whether age, the side affected by BPPV or the canal affected by BPPV had an association with abnormalities in BCV oVEMP testing in idiopathic BPPV (Table 5). Logistic regression analysis showed that only age had a significant positive association with abnormalities in BCV oVEMP testing [*p* = 0.0109 (age), *p* = 0.598 (side affected by BPPV), *p* = 0.576 (canal affected by BPPV)]. The odds ratio of abnormal BCV oVEMPs for the elderly group compared with the non-elderly group, for the affected side compared with the unaffected side, and for LSCC BPPV compared with PSCC BPPV was 2.676 (95% CI, 1.254–5.079), 0.831 (95% CI, 0.417–1.656), 0.815 (95% CI, 0.397–1.672), respectively.

**Table 5:** Binomial logistic regression analyses for investigating the association between abnormalities in BCV oVEMP testing and age, the side affected by BPPV and the canal affected by BPPV in idiopathic BPPV patients.

**Variable**	**Odds ratio (95% CI)**	***p*-value**
**AGE**		
Elderly group	2.676 (1.254–5.709)	0.0109*
Non-elderly group	Reference
**SIDE AFFECTED BY BPPV**		
Affected side	0.831 (0.417–1.656)	0.598
Unaffected side	Reference
**CANAL AFFECTED BY BPPV**		
LSCC	0.815 (0.387–1.672)	0.576
PSCC	Reference

BCV oVEMP, ocular vestibular evoked myogenic potential to bone-conducted vibration; BPPV, benign paroxysmal positional vertigo; SCC, semicircular canal; SE, standard error; CI, confidence interval; PSCC, posterior SCC; LSCC, lateral SCC;

* significant difference (*p < 0.05)

Collectively, the elderly group had a significant positive association with abnormal utricular function detected by BCV oVEMP testing, but it had no significant association with abnormal saccular function detected by ACS cVEMP testing or abnormal LSCC function detected by caloric testing. Utricular function was affected more by aging than by the side affected by BPPV or by the canal affected by BPPV.

## Discussion

We revealed that the elderly group (aged ≥65 years) had a significant positive association with utricular dysfunction in idiopathic BPPV patients, but there was no significant association with saccular dysfunction and the LSCC dysfunction. Utricular function was affected more by aging than by the side affected by BPPV or by the canal affected by BPPV.

In BPPV it is thought that fragments of otoconia are dislodged from the maculae of the otolith organ and enter the SCC duct (7). The increased prevalence of BPPV with age may be associated with age-related degenerative changes of the otoconia, calcium carbonate crystals in the otolith organ. In a laboratory study, the degeneration of otoconia and linking filaments with otoconial fragments were observed in aged rats (31). Furthermore, the possible existence of a relationship between BPPV and osteoporosis/osteopenia, the most common skeletal disorder in the elderly, has been reported (32, 33). A proposed underlying mechanism for the higher prevalence of BPPV in the elderly is a reduced capacity to dissolve the dislodged otoconia due to raised calcium levels in the endolymph in elderly patients with osteoporosis/osteopenia (32). Nevertheless, the reason for the increased prevalence of BPPV with age remains controversial.

The otoconia dislodgement might have an association with damage to the otolith organ (9). With the development and clinical application of VEMP testing, it became possible to evaluate the function of the otolith organs, and reveal the characteristics of otolith organ dysfunction. Dysfunction of the otolith organs in BPPV revealed by abnormalities in cVEMPs or oVEMPs is sometimes reported only on the side affected by BPPV (10, 12), and sometimes on both the affected and unaffected sides of BPPV (11, 34). Our group investigated the clinical features of patients with otolith organ-specific vestibular dysfunction (OSVD) who showed normal caloric responses and normal findings in video head impulse testing in each SCC plane, but abnormal responses in cVEMP and/or oVEMP testing (23). The most common diagnosis for the OSVD patients was BPPV. These findings strongly suggest an association between BPPV and otolith organ dysfunction. Our study revealed that utricular dysfunction was affected more by aging than it was by the affected side or canal of BPPV. From the results of this study, it can be inferred that BPPV is common in the elderly because they might suffer more damage to the utricle where the dislodgement of the otoconia mainly occurs.

Previous research into the effects of age on VEMPs in healthy individuals has revealed that the response rate of ACS cVEMPs decreases with age, although the manner in which the response rate decrease progresses is unclear, with reports of a gradual decline starting at 50 years old (16) and rapid decline over 65 years old (14). Another report showed that higher odds ratio of ACS cVEMP loss by age over 70 years old (35). On the other hand, there was also a report that the response rate of ACS cVEMPs showed a high rate, of 90% or more, even over 60 years old (36). The response rate of BCV oVEMPs decreases with age, although the manner in which the response rate decrease progresses is also unclear. One report showed significant reduction in response rate for BCV oVEMPs in subjects over 60 years old (37). On the other hand, another report showed that odds ratio of BCV oVEMP loss by age was not so high in 70–79 years old in comparison with under 70 years old (35). Regarding the effect of aging on VEMP amplitudes, it has been widely reported that the amplitude of cVEMPs and oVEMPs decreases with age (15, 16, 35). From these previous reports, it can generally be considered that abnormalities in both ACS cVEMPs and BCV oVEMPs increases with age. With regard to the effect of aging on morphological change in the otolith organs, it has been reported that the saccule is more affected by aging than the utricle in terms of hair cell degeneration and volume loss in the macular epithelia (38). In our study, the elderly BPPV group had a significant positive association with abnormal BCV oVEMPs. It also tended to show abnormal ACS cVEMPs but there was no significant association. It is probable that the dysfunction of the utricle in the elderly and the dislodgement of the utricular otoconia is the dominant characteristic in the pathogenesis of BPPV.

Our study showed that age, the affected side or affected canal of BPPV did not have a significant association with abnormalities in caloric testing. There are some reports that ipsilateral caloric hypoexcitability occurs in LSCC BPPV patients (39, 40). This hypoexcitability improved after the successful treatment of BPPV and might be explained by functional plugging of the lateral SCC by the dislodged otoconia (40). On the other hand, canal paresis has also been found in some patients with PSCC BPPV (41, 42). Caloric hypoexcitability in BPPV may not be affected by the affected canal of BPPV.

This study has several limitations. This is a retrospective data collection study, and information bias and selection bias cannot be excluded. Since the present study was performed at a university hospital, there may be a selection bias toward BPPV patients that were difficult to treat. Some of the patients who visited our balance disorder clinic and were diagnosed with BPPV did not perform all three of the vestibular function tests, these patients could not be included in the present study.

## Conclusions

In conclusion, we have revealed that aging is a risk factor for utricular dysfunction in idiopathic BPPV. On the other hand, aging has no significant association with abnormal saccular function or abnormal LSCC function. Utricular function is affected more by aging than by the side affected by BPPV or by the canal affected by BPPV.

## Author Contributions

CF conceived of the study, conducted the experiments, wrote the manuscript, and edited the manuscript for content. TK provided statistical advice, performed statistical analysis, and edited the manuscript for content. MK, YK, and KS conducted the experiments. MY and TY conceived of the study. SI conducted the experiments and edited the manuscript for content. TM conceived of the study and edited the manuscript for content.

### Conflict of Interest Statement

The authors declare that the research was conducted in the absence of any commercial or financial relationships that could be construed as a potential conflict of interest.
